# Blood-Vessel Mimicking Structures by Stereolithographic Fabrication of Small Porous Tubes Using Cytocompatible Polyacrylate Elastomers, Biofunctionalization and Endothelialization

**DOI:** 10.3390/jfb7020011

**Published:** 2016-04-20

**Authors:** Birgit Huber, Sascha Engelhardt, Wolfdietrich Meyer, Hartmut Krüger, Annika Wenz, Veronika Schönhaar, Günter E. M. Tovar, Petra J. Kluger, Kirsten Borchers

**Affiliations:** 1 Institute of Interfacial Process Engineering and Plasma Technology IGVP, University of Stuttgart, Stuttgart 70569, Germany; biggihuber@gmx.de (B.H.); annika.wenz@igvp.uni-stuttgart.de (A.W.); guenter.tovar@igvp.uni-stuttgart.de (G.E.M.T.); 2 Rheinisch-Westfälische Technische Hochschule Aachen, RWTH Aachen, Aachen 52074, Germany; sascha.engelhardt@rwth-aachen.de; 3 Fraunhofer Institute for Applied Polymer Research IAP, Potsdam 14476, Germany; wolfdietrich.meyer@iap.fraunhofer.de (W.M.); hartmut.krueger@iap.fraunhofer.de (H.K.); 4 Fraunhofer Institute for Interfacial Engineering and Biotechnology IGB, Stuttgart 70569, Germany; veronika.schoenhaar@igb.fraunhofer.de (V.S.); petra.kluger@igb.fraunhofer.de (P.J.K.); 5 Process Analysis & Technology (PA&T), Reutlingen University, Reutlingen 72762, Germany

**Keywords:** stereolithography, artificial blood vessel, small branched and porous tubes, biofunctionalization, thio-modified heparin, endothelialization

## Abstract

Blood vessel reconstruction is still an elusive goal for the development of *in vitro* models as well as artificial vascular grafts. In this study, we used a novel photo-curable cytocompatible polyacrylate material (PA) for freeform generation of synthetic vessels. We applied stereolithography for the fabrication of arbitrary 3D tubular structures with total dimensions in the centimeter range, 300 µm wall thickness, inner diameters of 1 to 2 mm and defined pores with a constant diameter of approximately 100 µm or 200 µm. We established a rinsing protocol to remove remaining cytotoxic substances from the photo-cured PA and applied thio-modified heparin and RGDC-peptides to functionalize the PA surface for enhanced endothelial cell adhesion. A rotating seeding procedure was introduced to ensure homogenous endothelial monolayer formation at the inner luminal tube wall. We showed that endothelial cells stayed viable and adherent and aligned along the medium flow under fluid-flow conditions comparable to native capillaries. The combined technology approach comprising of freeform additive manufacturing (AM), biomimetic design, cytocompatible materials which are applicable to AM, and biofunctionalization of AM constructs has been introduced as BioRap^®^ technology by the authors.

## 1. Introduction

New materials for additive manufacturing (AM) methods are gaining more and more interest for applications in medical engineering and medicine. Computer-based AM techniques can directly transform digital data into physical objects through layer-by-layer material deposition or layerwise material solidification. One particular focus is on photo-curable polymers for laser-based additive assembly of complex or individualized three-dimensional (3D) structures. In recent years, AM has already been utilized for assembling patient specific models from 3D images acquired by computer tomography or surface scanning enabling surgeons to plan complex interventions, to pre-adjust implants or to fabricate individualized surgical guides [1,2]. Future applications will increasingly comprise the fabrication of biocompatible and biofunctional scaffolds for the development of tissue models and implants with an individual shape [3]. Blood vessel reconstruction is of particular interest for both the development of *in vitro* models and artificial vascular prostheses. While the material specification list for blood vessel replacement *in vivo* is long and extremely challenging [4], the basic requirements for tubular supply systems for 3D tissue engineering are permeability and cytocompatibility. The inability to provide a sufficient and functional blood vessel system today is one of the main limitations in obtaining thick viable tissue models with the potential of mimicking the complex functions of native tissue [5]. With regard to the supply of several cubic millimeters of cells in a 3D matrix, tubular systems with single inlet, multiple branches, single outlet, and adjustable geometry are required. Endothelial cell adhesion and monolayer formation at the inner tube surface are additional prerequisites for simulating the controlled exchange of substances across the endothelial barrier. Fabrication of porous tubes has been realized earlier, e.g., by freeze-drying of biopolymers [6] or by electro-spinning of a broad range of materials [7,8]. Extrusion of bio-based materials and blends using coaxial nozzles also resulted in hollow fibers, which can eventually be perfused [9,10]. As drawback, such methods cannot produce branched structures. With shaping techniques, such as nanoimprint lithography or microcontact printing, more complex and branched microfluidic channels have been created [11,12,13]. However, the micro-channels thus produced are limited in application in engineering larger tissues [11,14]. The newly emerging AM techniques are now increasingly implemented to fabricate perfusable structures in a broad range of scales. We have recently reviewed current approaches to (bio-)printing of artificial blood vessels [15]. The most straightforward approach to perfusable tissue supply systems is the generation of a network of interconnected channels within the tissue matrix [16,17,18]. This has been achieved by extrusion-based deposition of branched networks of sacrificial materials such as carbohydrates, which were removed after a crosslinked cell-laden matrix had been added. Alternatively, hydrogel-based tubular structures with very high aspect ratios have, e.g., been fabricated by assembly of microspheres of crosslinked alginate, which formed by ejection of alginate droplets into a solution of CaCl_2_ [19,20]. Complex geometries such as that of branched blood vessels are now increasingly implemented in various scales by the newly emerging AM techniques. One major challenge is to support free-standing structures during buildup. Branched geometries have been assembled by the combined deposition of building materials and support materials [21,22]. The dimensions of such model structures are still in the centimeter range. Laser-based methods, e.g., stereolithography, two-photon-polymerization or digital light processing, induce solidifying of a photo-curable resin by layerwise selective crosslinking, and thus avoid the need of additional support. Such techniques have also been used to generate very tiny branched structures [23] or microstructured vessel walls [24] using acrylated polymers. Thus, obviously, the use of AM techniques has brought us closer towards the construction of perfusable tissue models and provides an alternative to the use of decellularized biological donor scaffolds. Consequently, there is a comprehensive need for new biomaterials capable of being applied in AM processes.

We have previously developed new material formulations which can be used for AM applications and tissue culture due to photo-reactivity, cytocompatibility, adjustable viscosity and the ability to tune the elastic properties of the cured materials [23,25,26]. However, synthetic materials that have been primarily adjusted to be processed in AM techniques are not necessarily cytocompatible or support cell adhesion. In this study, we suggest using thio-modified heparin and cysteine-coupled arginine-glycine-aspartic acid (RGDC) peptides for biofunctionalization of polyacrylate surfaces. We introduce the stereolithography-based method to fabricate porous tubes and branched tubular systems with 17 mm total length, 12 mm total width, and defined 100 µm pores from a novel acrylate based resin. A rinsing protocol was established to achieve cytocompatibility of the photo-cured material. Further, a seeding procedure with endothelial cells in a fluid-flow bioreactor with shear rates comparable to native capillaries was established for endothelial monolayer formation on the tube’s luminal surface *in vitro*.

## 2. Results

### 2.1. Post-Curing Treatments of UV-Cured PA

We developed an acrylate resin that is applicable in printing processes as well as for laser-based curing by stereolithography. We investigated UV-cured films in terms of post-curing treatments. Post-curing comprised a rinsing protocol in ethanol (EtOH, 70%) to achieve PA cytocompatibility, PA immersion within physiological buffer solution (PBS), and gamma (γ)-sterilization. Cytocompatibility testing was performed in a general way using fibroblast cells as recommended in DIN ISO 10993-5 [27]. Compatibility with endothelial cells was further confirmed by the seeding experiments of the material described in Section 2.4.

**Figure 1:**
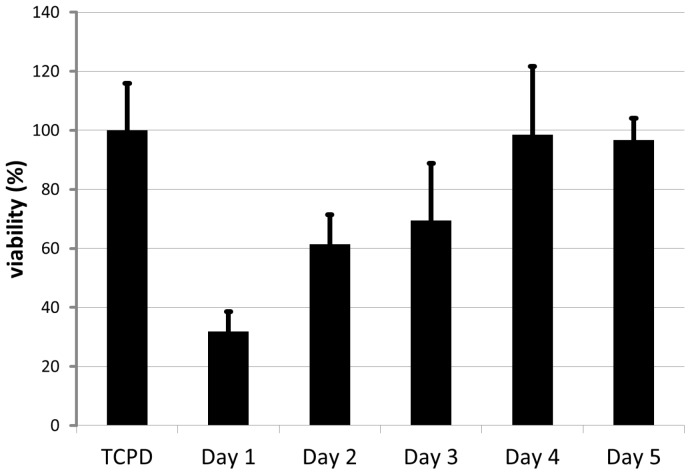
Viability of human fibroblasts cultured with material extracts for 24 h.

Rinsing in EtOH (70%) for five days increased the cytocompatibility of UV-cured PA from 31% ± 6.7% fibroblast viability on day one to 97% ± 7.2% on day five in relation to standard tissue culture petri dish (TCPD) substrates (Figure 1). Subsequent removal of EtOH by evacuation was crucial to achieve the cytocompatibility.

Cured polyacrylate (PA) was rinsed in EtOH (70%) for one to five days. Extracts from every material sample were tested according to DIN ISO 10993-5. The cell viability of PA was comparable to those cultured on tissue culture petri dishes (TCPD) after washing for four and five days.

A slight mass loss of 0.48% ± 0.22% was measured after the washing procedure of the PA material indicated the removal of soluble compounds. The immersion of UV-cured PA in EtOH (70%) hardly changed the Young’s modulus of the dried material (~18 MPa), while tensile strength dropped from 2.9 ± 0.4 MPa to 1.7 ± 0.1 MPa (Table 1). Immersion of UV-cured PA in PBS resulted in an increase of the Young’s modulus and tensile strength from 18.8 ± 1.7 MPa to 24.6 ± 2.3 MPa and from 2.9 ± 0.4 MPa to 3.5 ± 0.1 MPa, respectively. After γ-sterilization, the modulus and tensile strength rose from 18.8 ± 1.7 MPa to 21.3 ± 2.1 MPa and from 2.9 ± 0.4 MPa to 3.2 ± 0.5 MPa, respectively.

### 2.2. Production of Tubular Structures by Stereolithography

**Figure 2:**
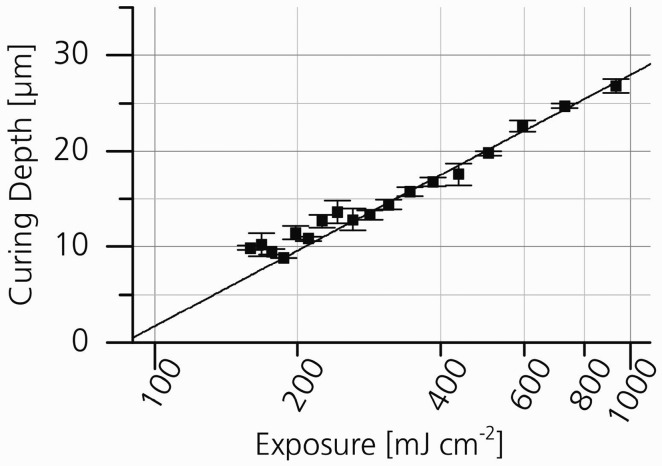
Curing depth plotted as a function of exposure. The smallest achievable curing depth for the PA resin with the used setup was approximately 10 µm.

**Figure 3:**
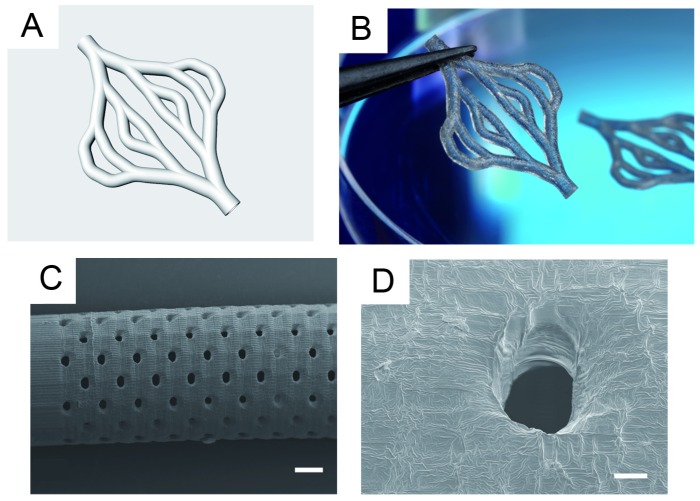
SLA-produced porous and branched tubular structures: (**A**) 3D model of a branched tubular structure, kindly provided by the University of Loughborough. (**B**) SLA fabricated tubular structure according to the design shown in (A). The inner diameter of the smallest branches is 1 mm the wall thickness is 300 µm. The complete construct is 12 mm wide and 17 mm high. (**C**) SEM image of a linear porous tube (scale bar: 400 µm). The inner diameter of the tube is 2 mm, the wall thickness is 200 µm and the total length is 30 mm. (**D**) Magnification of a single pore (scale bar: 40 µm). The diameter is approximately 100 µm spanning the entire wall crossection of 200 µm.

For the fabrication of branched and porous tubular structures a protocol for stereolithographic (SLA) curing of the resin was developed and the curing depth for the SLA process using the new PA resin was minimized. The curing depth $C_{D}\;$was determined at a fixed mean laser power of 25.4 mW. Variation of scan velocities between 50 mm s^−1^ to 410 mm s^−1^ resulted in exposure between 160 mJ cm^−2^ and 1307 mJ cm^−2^. When plotted in semi-logarithmic scale, the working curve followed the expected linear relationship (Figure 2). The minimum curing depth was approximately 10 µm and increased up to 30 µm for higher exposures. For small exposures, *i.e.*, high scanning speeds, the experimental data slightly deviated from the linear correlation. Based on these results, the layer separation distance was set to 20 µm to 30 µm for the generation of 3D tubular structures. The SLA process was then used to assemble linear tubular structures, branched tubular structures, and tubular structures with defined pores (Figure 3). The total length of the linear tubes was 30 mm, the inner diameter was 2 mm and the wall thickness was 200 µm. The pore diameter was 100 µm over the full wall thickness. The length of the branched tubular structures from inlet to outlet was 17 mm, the inner diameter was 1 mm and the wall thickness was 300 µm.

### 2.3. PA Surface Functionalization with Thio-Modified Heparin and RGDC-Peptides

**Figure 4:**
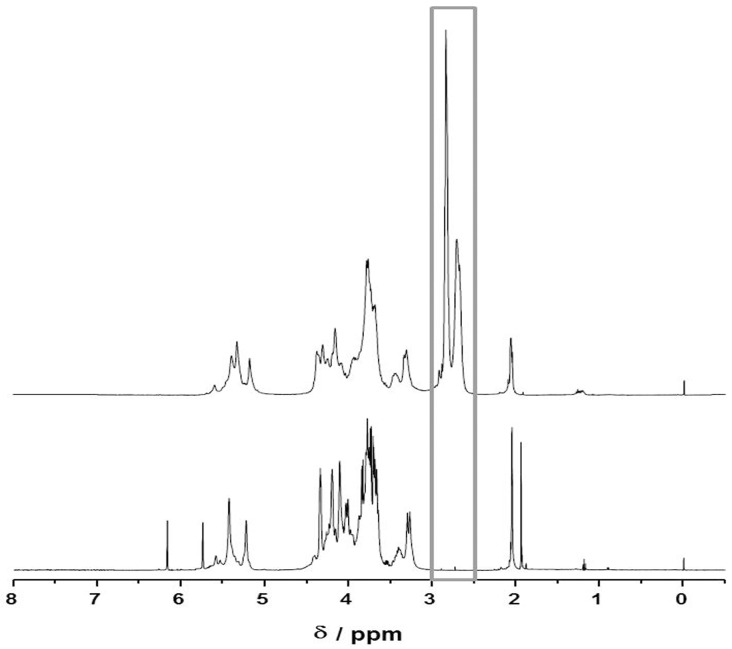
1H-NMR spectra of heparin (**bottom**) and thio-modified heparin (**top**). Signals at the chemical shift of δ = 2.65 ppm and the chemical shift of δ = 2.85 ppm correspond to methylen protons of the thio-functional linker.

With regard to providing model systems for small blood vessels we considered the integration of an endothelial monolayer. Thio-modified heparin and cysteine-coupled RGD were applied in order to equip the PA surfaces with cell recognition sites to enable the adhesion endothelial cells. Thio-modified heparin was synthesized by EDC mediated reaction of heparin with the dihydrazide linker DTPH and subsequent DTT mediated dissociation of the S-S bond. The successful derivatization of heparin was verified by ^1^H-NMR (D_2_O and 500 MHz). Figure 4 shows spectra of unmodified heparin and a thio-modified derivative. The resonances detected for thio-modified heparin at δ = 2.65 ppm and δ = 2.85 ppm were assigned to the insertion of two methylene groups by DTPH conjugation.

X-ray photon spectroscopy (XPS) was applied in order to investigate the chemical surface composition of pristine PA substrates and of PA substrates after incubation with aqueous solutions of the biomolecule derivatives. The results are summarized in Table 2. Sulfur (S) and nitrogen (N) signals were taken as proof for the presence of thio-modified heparin or RGDC peptides at the polymer surface because such elements were not present at pristine PA surfaces. Additionally, decreases of the atomic percentage of aliphatic carbon (C_ali_) indicated the formation of a surface coating, because aliphatic carbon was present mainly in the bulk polymer but only at low ratios in heparin and RGDC. The unchanged percentage of C_ali_ in untreated PA and PA treated with RGDC together with the absence of S or N indicated that the amount of peptides present at the surface was below the detection limit of XPS or that direct coupling of RGDC to the PA surface was not successful at all.

In contrast, at PA surfaces treated with thio-modified heparin or thio-modified heparin and RGDC, S and N were clearly detected. The relative amounts of S detected after functionalization with thio-heparin and functionalization with thio-modified heparin/RGDC was 0.3 at.% and 0.4 at.%, respectively, not indicating any significant changes of the S content upon RGDC coupling. The relative amount of N increased to 0.6 ± 0.1 at.% upon functionalization with thio-modified heparin and to 1.1 ± 0.1 at.% upon RGDC coupling. The approximated C1_ali_:N:S ratio of double disaccharide units of heparin is in the range of 0:2:(1–6) depending on the degree of sulfation and thio-modification. For the RGDC peptide the ratio is 2:8:1, thus the prominent increase of the N signal compared to the smaller increase of the S signal indicated the coupling of RGDC peptides to the thio-heparin coated surfaces.

### 2.4. Endothelial Cell Culture on Planar and Tubular Substrates

**Figure 5:**
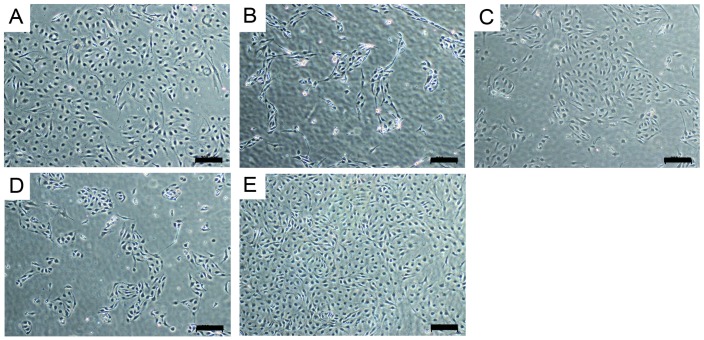
Light microscopic images of Human dermal microvascular endothelial cells (HDMECs) seeded on various surfaces for 24 h: (**A**) Tissue culture petri dish TCPD as control surface; (**B**) Untreated PA; (**C**) Polyacrylate (PA) functionalized with thio-modified heparin; (**D**) PA treated with cysteine-coupled arginine-glycine-aspartic acid (RGDC); and (**E**) PA functionalized with thio-modified heparin/RGDC (scale bar: 200 µm). Only few cells attached to the non-functionalized PA as well as on the PA treated with thio-modified heparin or RGDC. The functionalization with thio-modified heparin/RGDC resulted in a confluent cell monolayer comparable to the TCPD.

The adhesion and proliferation of human dermal microvascular endothelial cells (HDMEC) on planar material samples of pristine PA and functionalized PA were investigated after 24 h (Figure 5). On PA coated with thio-modified heparin and RGDC, a confluent cell monolayer formed comparable to that on TCPD. On non-functionalized PA and on surfaces that had been treated with the RGDC peptides or the thio-modified heparin separately, non-confluent cell colonies were found.

**Figure 6:**
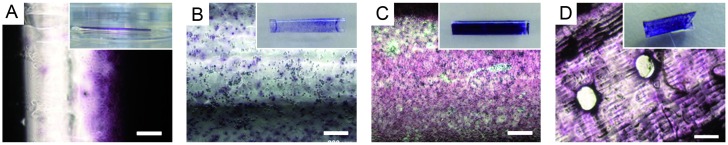
Endothelialization of tubular PA substrates. (**A**) static seeding led to non-confluent monolayers on one side of the tube; (**B**) rotating seeding of non-functionalized PA tubes resulted in low cell attachment all over the tube; (**C**) rotating cell seeding of tubes functionalized with thio-modified heparin/RGDC resulted in confluent monolayers on the inner surface; and (**D**) for the seeding of porous materials, an improved protocol was used. Scale bar: 200 µm.

For seeding HDMECs to the luminal walls of PA tubes, static and rotating setups were compared. Static incubation of cell suspension within the tube lumen resulted in cell attachment exclusively at the tubes’ bottom and thus in inhomogeneous cell layers. When the non-porous tube was rotated for 4 h during cell attachment homogenous endothelial cell adhesion to the complete inner tube wall was achieved. Cell attachment was generally lower on non-functionalized tubes than on biofunctionalized PA tubes (Figure 6). For porous tubes, the seeding procedure was modified. After 1 h rotation, cells were further allowed to proliferate for four days while cultured statically to compose a confluent monolayer in porous tubes. Assumingly, this was necessary because part of the cells were lost due to transiting the porous tube walls.

**Figure 7:**
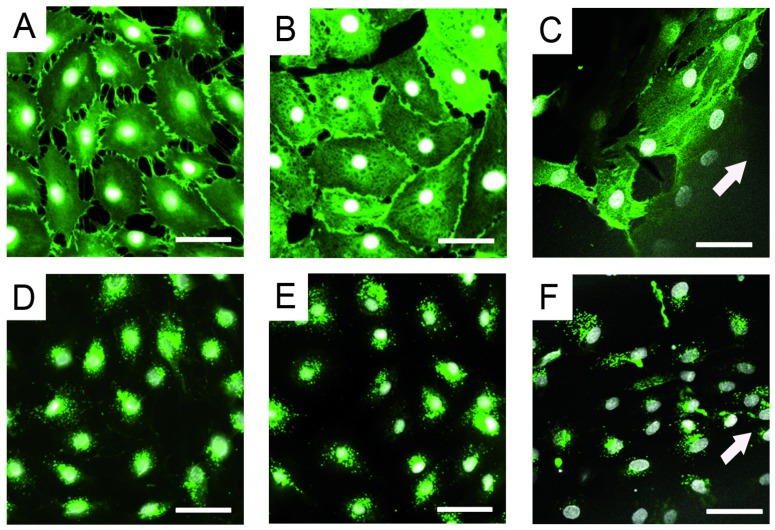
Expression of endothelial cell specific markers CD31 and vWF on thio-modified heparin/RGDC functionalized PA.

The effectiveness of cell seeding to the inner wall of polyacrylate tubes was compared applying the cell suspension in a static way or by rotating the tube at 0.5 rpm for 4 h at 37 °C. Cell density and viability was detected by MTT for: (A–C) non-porous tubes; (D) porous tube with pore size of approximately 200 µm. Cells were allowed to attach while the tube rotated for 1 h at 0.5 rpm and 37 °C. Subsequent static culture for four days allowed cell spreading and proliferation to create a confluent monolayer. Scale bar: 200 µm. After cell attachment, cells were cultured in a fluid flow bioreactor for seven days. The flow speed of the medium was raised slowly until the resulting wall shear stress of 1 N m^−2^ was achieved after 3.5 days, which is also experienced by endothelial cells in native capillaries [28]. We observed that endothelial cells aligned in the direction of the medium flow and changed their morphology to an elongated shape, while control cells cultured on TCPD conserved the cobblestone-like morphology, which is commonly observed in endothelia in static cultures. Specific markers for endothelial cells, CD31 and vWF, were expressed in both, the static and the dynamic regime (Figure 7).

HDMECs expressed CD31 and vWF when cultured under static and dynamic conditions: (A–C) CD31; (D–F) vWF; (A,D) static culture of HDMECs on TCPD (control); (B,E) static culture of HDMECs on PA functionalized with thio-modified heparin/RGDC; and (C,F) dynamic culture of HDMECs for seven days in a tubular PA material functionalized with thio-modified heparin/RGDC. Arrow shows the direction of flow (scale bar: 50 µm).

### 2.5. Characterization of Hemolytic Potential and Platelet Adhesion 

**Figure 8:**
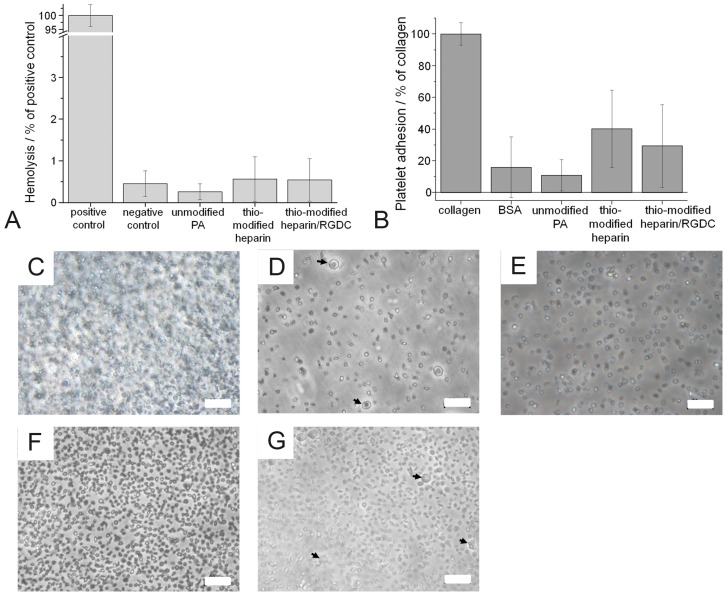
Hemolysis and platelet adhesion: (**A**) Hemolysis of erythrocytes on pristine and functionalized PA. Blood of three donors was tested on the materials in triplicates, and the results are shown as percentage of a positive control (erythrocytes lysed in water). For all tested materials the observed hemolytic activities were in the range of the negative control and ˂ 1% in relation to the positive control. (**B**) Platelet adhesion to the various surfaces. The results are shown as percentage of LDH activity detected after lysis of adhered platelets, the positive control was the LDH activity of platelets adhered to collagen type I surfaces and was set to 100%. BSA-coated TCPD served as negative control. (**C**) Platelets adhered to collagen type I; (**D**) BSA-coated TCPD; (**E**) unmodified PA; (**F**) PA functionalized with thio-modified heparin; and (**G**) PA functionalized with thio-modified heparin/RGDC. Activated platelets are marked with an arrow. Scale bar: 20 µm.

With regard to future uses of endothelialized surfaces in direct contact with blood, we preliminary addressed hemocompatibility of PA and coated surfaces in terms of hemolysis and platelet adhesion. The hemolytic activity of all surfaces tested was below 1% of the positive control and none of the surfaces provoked any hemolysis significantly different from the negative control (Figure 8).

Light microscopic surface analysis revealed that the positive control surface (collagen type I) was densely covered with platelets, while considerably fewer platelets were found on the negative control (BSA-coated TCPD) and unmodified PA (Figure 8). On the surfaces functionalized with thio-modified heparin and thio-modified heparin/RGDC, more platelets adhered than to the unmodified PA. Very few activated platelets were observed. The relative quantification of adhered platelets was achieved by quantification of the amount of released LDH after platelet lysis (Figure 8). The results confirmed that platelet adhesion to unmodified PA (10.9% ± 9.8%) was similar to the negative control (16.9% ± 19.1%). Significantly higher numbers of platelets adhered to the coatings of thio-modified heparin (40.2% ± 23.6%). The adhesion to surfaces coated with thio-modified heparin/RGDC seemed to be at an elevated level (29.4% ± 26.0%) though not significant.

## 3. Discussion

Additive manufacturing methods are gaining importance in the fabrication of small-caliber artificial vascular grafts. Currenlty, no appropriate material is available that can be used in biological applications and in medical engineering in medicine. In this study, we investigated means to achieve cytocompatibility and biofunctionality of a new material formulation which was used to construct porous tubular structures with photo-curing based additive assembly by stereolithography.

### 3.1. Post-Curing Treatments of UV-Cured PA

We observed decreasing cytotoxicity upon extensive immersion EtOH (70%) suggesting that soluble substances with elevated toxicity, e.g., the photoinitiator Irgacure 184 which has been shown to be cytocompatible at low concentration (˂ 0.02% [29,30]) or unreacted monomers remained within the polymer network after UV crosslinking and were then removed during rinsing. This observation was accompanied by a mass loss of 0.5% of the dried polymers before and after rinsing with EtOH. A moderate decrease of the Young’s modulus and a strong dropping in tensile strength was also observed after the washing procedure with EtOH (70%) while a rise in the Young’s modulus was observed upon exposure to PBS. We assume that microscopic destruction of the polymeric network occurred due to an elevated degree of swelling in EtOH. In spite of the hydrophobic properties of PA we detected a small uptake of 1% to 2% (w/w) water into the polymer bulk material. Therefore, we propose that in the case of PBS the swelling effects resulted in an expanded (but not destructed) network with higher mechanical values.

The increase in modulus and tensile strength after γ-irradiation is assumed to be the result of increased degree of crosslinking of the polymer precursor molecules. The γ-radiation consists of higher energy and ingresses deeper into the material than UV radiation. Therefore, additional crosslinking of remaining monomers and elevated strength of the material has to be taken into account for γ-irradiated material in comparison to non-treated PA.

The elastic properties of native arteries are highly complex (anisotropy, viscoelasticity, and non-linear stress-strain relationship) and currently unmet by the use of synthetic materials. The material presented here was rather intended to provide adequate handling features required for mounting the perfusion systems into the bioreactor, than as proper mechanical model for blood vessels. The mechanical testing results showed that the solid cured-PA was sufficiently elastic and durable but surmounted the elasticity of natural blood vessels of around 2 MPa only to some extent [31]. The properties of porous PA tubes will be characterized and adjusted in additional studies in terms of their mechanical answer to pulsed blood pressure and physical stretching.

### 3.2. Production of Tubular Structures by Stereolithography

The vertical resolution is a critical value for the generation of tubular structures by SLA. It is determined by the curing depth, which depends on the penetration depth of the employed wavelength within the photosensitive material. Thus, the curing depth can be minimized by increasing the absorption cross-section of the photosensitive material or by reducing the exposure from the SLA apparatus by, e.g., increasing the scanning speed. In our study, reduction of exposure and thus reduction of crosslinking density resulted in insufficient mechanical strength for the generation of 3D structures. Consequently, 2,2'-dihydroxy-4,4'-dimethoxy-benzophenon was added as an absorber to increase the absorption cross section of the photosensitive material. For this resin formulation the exposure and the curing depth showed the expected linear correlation on a semi-logarithmic scale. The deviation from the linear correlation for small exposures, *i.e.*, high scanning speeds was most probably due to the fact that at a pulse repetition rate of 20 kHz the pulse overlap was decreasing for the upper scan speeds. The lateral distance between two laser pulses at a scan speed of 400 mm s^−1^ was 20 µm and therefore almost as large as the beam waist, such that the curing depth reached the minimum.

The freeform capabilities of the SLA process can be used to incorporate additional structural functionalities into the 3D tubular structure. For the generation of artificial supply systems for biological tissue models we chose to develop a hydrophobic material composition in order to avoid extensive swelling and thus geometrical changes of the tubes within aqueous environments. As a consequence, the vessel walls had to be porous in order to allow the free exchange of water and nutrients into and from surrounding tissue as well as the passage of cells, e.g., for neoangiogenesis. We demonstrated that the presented material process combination allowed for fabrication of tubular structures with well controlled 100 µm pores.

In the future, the pore size must be reduced to 20 µm or smaller to allow endothelial cells to cover the pores and to achieve a physiological barrier controlled by the endothelial layer [32,33]. Currently, higher resolutions required reduced laser intensities and thus resulted in decreased mechanical stabilities. In order to overcome such hurdles, further tuning of material properties and curing parameters will be addressed in future studies.

### 3.3. PA Surface Functionalization with Thio-Modified Heparin and RGDC-Peptides

Endothelial cells are known to bind to proteins from the basement membrane (e.g., collagens, laminin, perlecan, and fibronectin), serum proteins (fibrin and fibrinogen) or the respective integrin-binding epitopes including RGD, YIGSR, and GFOGER [34,35,36]. Some studies reported that coating with heparin improved EC adhesion to surfaces. The interaction of surface bound heparin with the heparin-binding domain of fibronectin is discussed as one possible mechanism mediating cell adhesion [37], but the interaction of heparin with endothelial cells is not completely understood yet [38,39,40] and most studies heparin-based coatings alone only supported initial cell adhesion but cell loss and irregular cell shapes were observed after some time [37]. In order to achieve stable endothelial lining of the tube surfaces we combined heparin with RGD peptide coupling. We used thio-modified heparin and RGD-cysteine to modify the PA surfaces. PA surfaces eventually contain unreacted acrylate functions (C=C) after curing [26]. Such remaining double bounds (RDB) can be used for the conjugation of thio-functional molecules via thiol-Michael reactions. Although the covalent coupling of thio-functions to acrylate functions at the PA surface could not be deduced from XPS data directly, the increase in Sulfur (S) content and the change in N:S ratio at the PA surface indicated heparin immobilization and RGDC coupling, respectively. While in our hands the direct coupling of the cysteine to the PA surface was not efficient the thio-functionalized heparin polymers obviously mediated subsequent coupling of RGDC. We concluded that the thiolated polymer chains, once they bound to acrylic functions at the PA surface, served as multiplicator displaying a high number of thio-functions for RGDC coupling via disulfide formation.

### 3.4. Endothelial Cell Culture on Planar and Tubular Substrates

The effective contribution of short recognition sites such as RGD to cell adhesion in protein-rich environments as *in vivo* is discussed controversial [41]. However, *in vitro* RGD is one of the most often used cell recognition sequences. It was shown in several studies that RGD enhanced endothelial cell attachment [42,43,44,45]. In accordance with our own results other studies that combined heparin-based coatings with specific cell adhesion sequences such as fibronectin or RGD, the addition of cell adhesion sites stabilized or improved [37,38,40] the adhesion of endothelial cells.

In our hands rotating seeding led to a complete homogenous monolayer of endothelial cells on the heparin-RGD functionalized inner tube wall. We have shown that endothelial cells stayed attached and aligned along the media flow even after culturing them for 7 days under fluid flow, thereby applying the physiological wall shear stress of 1 N m^−2^ for 2 days. Previous studies had also shown that endothelial cells, e.g., adhered on RGD-functionalized expanded polytetrafluoroethylene grafts (ePTFE) and oriented in the direction of shear stress and formed a cell layer more resistant to shear stress compared to a fibronectin coating under a shear rate of 0.8 N m^−2^ for 24 h [46]. Other authors showed that cells adherent to a RGD-coated polyethylene terephthalate (PET) surface endured exposure to 12 dyn cm^−2^ (1.2 N m^−2^) shear stress, mimicking arterial conditions, for two hours [47]. A higher seeding efficiency was additionally observed when using endothelial progenitor cells on RGD-coated surfaces compared to human umbilical vein endothelial cells (HUVECs) [48].

### 3.5. Characterization of Hemolytic Potential and Platelet Adhesion

Although intended for *in vitro* use in its current state, we also assessed the new PA material and its coatings in a preliminary study with regard to hemolytic and platelet-adhesive effects. As expected, none of the surfaces showed hemolytic activities. However, thio-modified heparin as well as heparin/RGDC coated surfaces seemed to slightly mediate platelet adhesion in contrast to unmodified PA. It is known that contact activation of platelet integrins, e.g., by the RGD sequence generally leads to platelet adhesion, spreading and thrombus formation at surfaces [49,50,51,52]. Interestingly, some *in vivo* experiments also proofed reduction of platelet adhesion on RGD functionalized surfaces compared to unmodified polymers such as PCL [53].

Profound platelet adhesion to thiol-groups was also reported for thiolated surfaces that carried low surface charge densities [54]. It is discussed to be mediated by the existence of free thiol-groups on the surface of platelets [55,56]. The surfaces described here also allow for the assumption that masking of free thio-groups of thio-modified heparin has occurred by RGDC peptide coupling via disulfide formation and may have thereby reduced platelet adhesion. Still, the thrombogenic potential of the surface stayed on an elevated level consistent with the observation of other authors, who reported αIIbβ3 integrin mediated adhesion of platelets to the RGD sequence [52]. In contrast to the results of Corum *et al*. hydrophobicity of the untreated PA alone did not result in elevated platelet adhesion [54].

## 4. Experimental Section

### 4.1. Preparation of (co-BPA-co-IBA-co-IL)-Polyacrylate in Planar and Tubular Geometry

Bisphenol A ethoxylate diacrylate (*M* = 688 g mol^−1^) (BPA), lauryl acrylate (LA), and isobornyl acrylate (IBA) were purchased from Sigma Aldrich^®^; Irgacure^®^ 184 was used as photoinitiator (kindly provided by Ciba AG, Basel, Switzerland). First, 49.75% BPA, 37.31% IBA, 12.44% IL and 0.5% Irgacure^®^ 184 were blended (PA-resin) and used for sample preparation by curing with UV light under an argon atmosphere. The cured polyacrylate, a statistical (*co*-BPA-*co*-IBA-*co*-IL)-polyacrylate (PA), has been described recently [57].

#### 4.1.1. Planar Sample Preparation

The volume of 0.3 mL PA-resin was loaded into a polystyrene petri dish (diameter 35 mm), flushed with argon, and irradiated 4 min with full UV spectra (Bluepoint 4 ecocure, Hoenle, Germany: 40 mW cm^−2^ at 365 nm).

#### 4.1.2. Tubular Sample Preparation by Dip Coating

Small glass rods (*d* = 2 mm, *L* = 75 mm; Marienfeld, Lauda-Königshofen, Germany) were dip coated manually into PA-resin, led drop off surplus material while smoothly turning the tubes between two fingers, placed in a glass beaker and UV cured for approximately 1 min while permanently flushed with argon. The procedure was repeated six times. Subsequently, these multilayers were flushed with argon and irradiated for 4 min. The PA coated glass rod was left in water overnight to separate the coating easily from the glass rod remaining PA tubes with 2 mm inner diameter.

#### 4.1.3. Tubular Sample Preparation by SLA

For the preparation of AM generated tubular samples an SLA setup was used. An UV laser source (FTSS 355-Q2, CryLaS GmbH, Berlin, Germany) emitting laser pulses with a pulse duration of approximately 1 ns at a wavelength of 355 nm was focused by a f-Theta lens with a focal length of 100 mm (JENar^®^ 03-70FT-100-355, JENOPTIK Optical Systems Inc., Jena, Germany). The laser focus can be translated using a scanner system (PS2-10, Cambridge Technology, Planegg, Germany) and yields a maximum working field of 52 mm × 52 mm. The resulting beam waist was measured by a beam analyzing camera (SP620U, Ophir Optronics Solutions Ltd., Darmstadt, Germany) to approximately 30 µm. For sample preparation, the laser source was set to a repetition rate of 20 kHz and the photosensitive resin was placed in a small volume vat. For SLA, 0.1% (w/w) of 2,2'-dihydroxy-4,4'-dimethoxy-benzophenon was added to the resin as an absorber to reduce the curing depth. The tubular structures were built in a layer-by-layer approach, with a layer separation distance of 20 µm. The curing depth $C_{D}\;$was determined for different exposures E applying the following equation [58]:

(1) $$C_{D}=D_{P}\times \text{ln}\left(\frac{E}{E_{crit}}\right)$$

where $D_{P}$ denotes the penetration depth at which the initial intensity is reduced to 1/e (~37%) and $E_{crit}$ is the critical exposure necessary to initiate polymerization.

### 4.2. Sterilization

Gamma (γ)-sterilization for the mechanical tests was performed by a certified commercial service provider (BBF Sterilisationsservice GmbH, Kernen-Rommelshausen, Germany) at approximately 30 kGy.

### 4.3. Characterization of Young’s Modulus and Tensile Strength of PA

Samples were cured using full spectra UV in a silicon bone template for 4 min under argon atmosphere and measured with Zwick/Roell 1445 (Zwick, Ulm, Germany). Samples were prepared with: (a) no further treatment; (b) after γ-sterilization; (c) washed for five days in 70% EtOH then dried in vacuum overnight at 40 °C; and (d) like (c), followed by exposing to PBS buffer for 24 h.

### 4.4. Evaluation of the Cytocompatibility

The cytocompatibility of post-cured PA was characterized according to DIN ISO 10993-5 [27]. For extract preparation planar PA samples were covered with 3 mL DMEM (Biochrom, Berlin, Germany) for 24 h at 37 °C under static conditions. Human dermal fibroblasts were seeded in 96-well tissue culture polystyrene plates (10^4^ cells per well). PA extracts were then pipetted onto the cell cultures (200 µL per well), 10% FCS per well was added and cells were incubated for 24 h at 37 °C. Cells incubated in DMEM were provided as a negative control. The cell viability was determined by WST-1 assay (Roche, Mannheim, Germany) according to the manufacturer’s protocol using a microplate reader (Infinite 200 Pro, Tecan, Crailsheim Germany). Mass loss (%) was calculated from tubular PA samples (*n* = 12). The vacuum dried mass was determined before and after extracting tubes with 70% EtOH for a period of five days:

(2) $$\%mass\;loss=\left(\frac{m{\left(PA\;tube\right)}_{after}}{m{\left(PA\;tube\right)}_{before}}\right)\times \;100$$

### 4.5. Synthesis of 3,3-Dithiobis(Propanoic Dihydrazide) (DTPH)

The synthesis was performed based on Vercruysse *et al*. [59]. Five grams of 3,3- dithiodipropionic acid were diluted in 40 mL absolute methanol with a few drops of oleum. The mixture was refluxed under argon for 1 h. The reaction mixture was concentrated to 10 mL volume, diluted in ethylacetate and washed with 1 M NaOH and water. The organic phase was dried in vacuum to yield the ester. Five grams of the product were dissolved in 40 mL ethanol (EtOH) and added to 15.75 g hydrazine hydrate. The solution was warmed up, stirred for 4 h, and the product was then precipitated through cooling to 4 °C. The filtered and washed crystals were dried under vacuum. 1H-NMR: (DMSO-d6) δ 9.07 (s, 2H, NH_2_–NH–CO–), 4.20 (s, 4H, NH_2_–NH–), 2.89 (t, 4H, S–CH_2_–), 2.41(t, 4H, –CH_2_–CO–).

### 4.6. Synthesis and Characterization of Thio-Modified Heparin

Heparin sodium salt was chemically modified following Shu *et al*. [60]. Briefly, 476 mg DTPH were dissolved in 20 mL double distilled water (ddH_2_O). Five hundred milligrams heparin (CAS 01.08.9041, Aldrich, Germany) and 192 mg 1-Ethyl-3-(3-dimethylaminopropyl) carbodiimid (EDC, CAS 25952-53-8, Fluka, Darmstadt, Germany) were added and the reaction mixture was stirred for 4 h at pH 4.75, then increased to pH 8. Then, 1.54 g dithiothreitol (DTT) were added and stirred overnight. The solution was dialyzed first in 0.3 M NaCl solution with 0.1% benzyl alcohol at pH 3.5, then in ddH_2_O with 0.1% benzyl alcohol at pH 3.5, and then in ddH_2_O. The purity of the product and the substitution of thio-groups were verified by 1H-NMR.

### 4.7. PA Surface Functionalization with Thio-Modified Heparin and RGDC-Peptides

For surface functionalization with thio-modified heparin or RGDC peptides, PA was incubated overnight with thio-modified heparin (2 mg mL^−1^) or RGDC (0.2 mg mL^−1^) in PBS buffer (pH 7.2) at room temperature (RT) on a shaker. Afterwards, the samples were washed three times with ddH_2_O. Sequential functionalization of PA surfaces with thio-modified heparin and RGDC via disulfide formation was performed according to Kirihara *et al*. [61]. After immobilization of thio-modified heparin, the samples were immersed at RT with 0.25 mg mL^−1^ RGDC, 0.125 nmol mL^−1^ potassium iodide (Fluka, Darmstadt, Germany) and 3% hydrogen oxide (14.7 µmol mL^−1^, Sigma-Aldrich, Germany) and shaken for 10 h. Subsequently, the samples were rinsed with ddH_2_O. For disinfection, all samples were incubated with 70% EtOH for 0.5 h before seeding of cells.

### 4.8. Endothelial Cell Culture on Planar and Tubular Substrates

#### 4.8.1. Cell Culture on Planar Substrates

Human dermal microvascular endothelial cells (HDMECs) were seeded at a density of 10^5^ cells on planar PA samples per dish (diameter: 35 mm) in endothelial cell growth medium (ECGMmv) media (PromoCell, Heidelberg, Germany) to evaluate cell adhesion and proliferation. Cells were incubated at 37 °C for 24 h and then analyzed by bright-field microscopy (Nikon, Düsseldorf, Germany).

#### 4.8.2. Cell Culture on Tubular Substrates

HDMECs were seeded to the inner walls of tubular scaffolds at 8 × 10^6^ HDMECs mL^−1^ using either a static set up or rotating the tube at 0.5 rpm for 1 h (porous tubes) or 4 h (non-porous tubes) at 37 °C. Porous tubes were then statically cultured for four days to allow cell proliferation.

For dynamic culture of HDMECs in tubes, a fluid flow bioreactor (Unitechnologies SA, Gals, Switzerland) was used. Upon adhesion, HDMECs were cultured experiencing a wall shear stress of 0.012 N m^−2^ for 24 h. The shear stress was then increased at a rate of 0.012 N m^−2^ h^−1^ up to the maximum of 1 N m^−2^, which is comparable to the shear stress experienced by endothelial cells in native capillaries [28].

Laminar wall shear rate was calculated as followed:

(3) $$\tau_{wall}=\frac{4\times \bar{v}\times \eta }{r_{i}}$$

where $\tau_{wall}$ in N m^−2^ is the wall shear rate, $\bar{v}$ in m s is the velocity of the fluid, η in Ns m^−2^ is the dynamic viscosity of the fluid and $r_{i}$ in m is the inner radius of the tube.

The cells were cultured for 48 h with a wall shear rate of 1 N m^−2^. During 7 days two media exchanges were performed.

### 4.9. Staining of Cells

For the detection of viable cells within the tubes, 3-(4, 5-dimethylthiazolyl-2)-2,5-diphenyltetrazolium bromide (1 mg mL^−1^; MTT; Sigma-Aldrich, Taufkirchen, Germany) in ECGMmv medium (PromoCell, Germany) was pipetted into the tubes and incubated for 45 min at 37 °C. For immunofluorescence staining of the endothelial cell marker CD31 and von Willebrand Factor (vWF) cells were fixed using 4% phosphate-buffered formaldehyde (Roth, Karlsruhe, Germany) for 15 min. Prior to blocking with 3% BSA/PBS for 30 min, cells for vWF staining were permeabilized using a PBS/0, 1% Triton-X-100 (Sigma-Aldrich, Taufkirchen, Germany) solution for 10 min at RT. Primary antibodies were diluted with antibody diluent (all DAKO, Germany; vWF: 1:600; CD31: 1:800) and incubated overnight at 4 °C. Then, Cy3-labeled secondary antibody (1:1000; Jackson Immuno Research, Suffolk, UK) was applied for 0.5 h. Nuclei were stained with DAPI (1:1000) for 15 min in the dark and cells were analyzed in PBS^+^ with a laser scanning microscope (Zeiss, Jena, Germany).

### 4.10. Hemolysis and Platelet Adhesion

Hemolysis and platelet adhesion were assessed as described in Motlagh *et al.* [62]. For hemolysis testing, heparinized blood from three healthy volunteers was used. All samples were probed with blood from each donor in triplicates. Briefly, 0.4 mL whole blood was diluted in 20 mL physiologic salt solution. One milliliter was pipetted onto each sample or into tissue culture petri dishes (TCPD; negative control) in triplicates. Complete lysis of the erythrocytes was achieved by addition of 10 mL sterile ddH_2_O to 0.2 mL donor blood (positive control on TCPD). All samples were incubated under gentle agitation for 2 h at 37 °C. Afterwards, the blood dilutions were centrifuged at 13,250 g for 10 min. Absorbance of released hemoglobin was measured at 541 nm in 96-well plates using a microplate reader (Infinite 200 Pro, TECAN, Crailsheim, Germany). The percentage of hemolysis was calculated as follows:

(4) $$\%Hemolysis=\frac{absorbance\;of\;test\;polymer}{absorbance\;of\;positive\;control}\times 100$$

For platelet adhesion testing, human platelet concentrates (HPC; leukocyte-depleted, each pooled from four donors) were purchased from the Institute for Transfusion Medicine of the Katharinenhospital (Klinikum Stuttgart, Stuttgart, Germany). Six independent experiments were conducted, each including all material samples in triplicates. Collagen gels (6 mg mL^−1^; Fraunhofer IGB, Stuttgart, Germany) on TCPD served as positive control, TCPD surfaces modified with BSA (40%) were used as negative control [63]. Cell counting of the HPCs resulted in counts between 7.97 × 10^6^ cells mL^−1^ and 8.79 × 10^6^ cells mL^−1^. In total, 250 µL cm^−2^ HPC were incubated on every sample at 37 °C for 1 h. The surfaces were carefully washed three times with PBS to remove non-adhered platelets. Light-microscopic pictures were taken (Nikon, Germany). Subsequently, the adhered cells were lysed with PBS/2% Triton-X-100 at 37 °C for 30 min. Lactate dehydrogenase (LDH) activity (in Unit L^−1^) was assessed from supernatants with the RX Daytona analyzer (Randox, Crumlin, United Kingdom). Platelet adhesion was calculated in correlation to the platelets that adhered to the positive control as follows:

(5) $$\%Platelet\;adhesion=\frac{LDH\;activity\;on\;tested\;sample}{LDH\;activity\;on\;collagen}\times 100$$

## 5. Conclusions

The presented material system consisting of a photo-crosslinkable polymer resin, which forms a cytocompatible elastic polymer upon UV curing, and a biofunctional coating contributes to the development of a new class of materials that fulfill a range of current demands associated with biomaterials. In particular, fabrication of complex structures comes more and more into focus, in addition to non-toxicity, sterilizability and the potential to achieve genuine biological surfaces, *i.e.*, endothelialization. In this study, we present a polyacrylate resin that can be photo-cured to generate scaffolds in various geometries using a photoinitiator and UV irradiation. We fabricated complex tubular structures with multiple branches and defined pores by stereolithography and achieved biofunctionalization and confluent endothelial lining of the inner tube walls within a flow bioreactor. In conclusion, we provide a technology platform that enables additive freeform fabrication of complex cytocompatible objects covering structure sizes from the µm range to cm range with biofunctional surfaces, and thus being promising for sophisticated scaffolding for *in vitro* tissue models and for individualized medical engineering.

## Figures and Tables

**Table 1:** Young’s modulus and tensile strength of PA after four min of UV-curing, before and after additional rinsing for five days in EtOH (70%), and after additional 24 h immersion in physiological buffer or after additional gamma sterilization.

PA Material	Young’s Modulus (MPa)	Tensile Strength (MPa)
UV (4 min)	18.8 ± 1.7	2.9 ± 0.4
UV (4min) + EtOH (70%) treatment and dried in vacuum	18.3 ± 0.4	1.7 ± 0.1
UV (4 min) + γ-sterilization	21.3 ± 2.1	3.2 ± 0.5
UV (4 min) + PBS buffer	24.6 ± 2.3	3.5 ± 0.1

**Table 2:** Results of XPS analysis of PA surfaces without treatment, after immersion with RGDC solution, solution of thio-modified heparin, or both biomolecules in sequence: 168.2 eV to 168.4 eV binding energy of sulfate-sulfur, 163.4 eV binding energy of sulfur from S–C bonds, 399.7 eV to 401.4 eV binding energies of nitrogen in amide bonds and amino functions, 284.6 eV binding energy of aliphatic C-C bonds.

Title	Untreated PA	RGDC	Thio-Modified Heparin	Thio-Modified Heparin/RGD
*Mean average/at.%*				
C1 (284, 6 eV)	49.4	50.3	46.6	45.0
S1 (168.2 eV to 168.4 eV)	0.0	0.0	0.2	0.2
S2 (163.4 eV)	0.0	0.0	0.1	0.2
N total	0.0	0.0	0.6	1.1
*Standard deviation/at.%*				
C1 (284, 6 eV)	2.5	0.1	0.1	0.5
S1 (168.2 eV to 168.4 eV)	0.0	0.0	0.0	0.0
S2 (163.4 eV)	0.0	0.0	0.0	0.1
N total	0.0	0.0	0.1	0.1
